# Variables with a negative impact on the quality of life of gays and lesbians in Spain: evaluation of homophobic and lesbophobic indicators to guide youth education

**DOI:** 10.3389/fpsyg.2024.1323208

**Published:** 2024-03-15

**Authors:** José-Rufino García-Sánchez, Francisco-Javier Gago-Valiente, María-José López-López, Adrián Segura-Camacho, Luis-Carlos Saenz-de-la-Torre, Emilia Moreno-Sánchez

**Affiliations:** ^1^Department of Pedagogy, Faculty of Education, Psychology and Sports Sciences, University of Huelva, Huelva, Spain; ^2^Center for Research in Contemporary Thought and Innovation for Social Development (COIDESO), University of Huelva, Huelva, Spain; ^3^Nursing Department, Faculty of Nursing, University of Huelva, Huelva, Spain; ^4^Department Clinical and Experimental Psychology, Faculty of Education, Psychology and Sports Sciences, University of Huelva, Huelva, Spain; ^5^Department of Social, Development and Educational Psychology, Faculty of Education, Psychology and Sports Sciences, University of Huelva, Huelva, Spain

**Keywords:** quality of life, homophobia, lesbophobia, public health, health care, social sciences

## Abstract

**Background:**

Despite the evidence about the negative impact of homophobic and lesbophobic attitudes on the quality of life of these individuals, the World Health Organisation (WHO) continues to report a lack of research and understanding regarding the health of gays and lesbians and LGTB people in general. There is a growing commitment in public health to understand and improve the health and well-being of LGTB people, and it is very important that professionals in social, educational and health care settings are adequately trained and informed to solve the problems that persist in this population. The aim of this study was to identify homophobic and lesbophobic attitudes in a Spanish youth population, analyzing the relationship of these attitudes with sociodemographic, cultural, political and personal variables.

**Methods:**

A descriptive, cross-sectional study was carried out with 325 young people aged 18–30 years. The information was collected through the Modern Homophobia Scale of Raja & Stokes, in which sociodemographic and sociocultural variables were also gathered. The data were analyzed and correlations were estimated.

**Results:**

The males showed a more negative attitude toward homosexual people compared to the females. The participants with a higher education level presented more positive attitudes toward homosexual people. Younger people with a heterosexual orientation had more negative attitudes toward homosexual people compared to those with a homosexual orientation. The participants with a stronger right-wing political tendency presented a greater percentage of negative attitudes toward LGTB people.

**Conclusion:**

Variables such as education level, sexual orientation and political ideology may have a significant influence on the attitudes toward homosexual men and/or lesbian women. Different results were obtained as a function of sex, thus it is important to consider the gender perspective in future studies that tackle this topic.

## 1 Introduction

The LGTB collective is a collective made up of lesbian, gay, bisexual, transgender, intersex, queer and others. This research will only focus on attitudes towards homosexual men and women, i.e., gays and lesbians.

One of the main contributions of the 20th century was the consolidation of the welfare state, which consists in the intervention of the public authorities in the economic and social life of the country, with the aim of correcting the functioning deficiencies of the market, redistributing the resources and creating a legal system for the protection of the citizen against the severe social problems that are generated in society (Briceño and Gillezeau, 2012).

When referring to the term welfare, it is important to remember that the WHO (Organización Mundial de la Salud, 2006), in its constitutive act of April 7th 1948, defined health as a complete state of physical, mental and social welfare, and not merely as the absence of affectations or diseases. From that date, this concept has not been modified. This means that both the planning and management of health by the different regimes of the welfare state must be based on prolonging such complete welfare state to its maximum extent and preventing its deterioration and disappearance.

The concept of quality of life is directly related to health and welfare (Mceberg, 1993). Fernández et al. (2001) defined health-related quality of life as the level of welfare derived from the person’s evaluation of different dimensions of her/his life, considering how these are influenced by her/his health state.

Some researchers (Guyat et al., 1993; Beckie and Hayduk, 1997) state that, in order to measure quality of life, it is necessary to focus, on the one hand, on purely objective variables, such as the biochemical parameters of the human body, and, on the other hand, on indicators proposed by social sciences, such as happiness, satisfaction with life and subjective welfare. However, WHO recommends the contemporary biopsychosocial model and approach to disease and health, which takes into consideration the dynamic interaction of its three components: biological, psychological and social.

Subjective welfare may present unfavourable results, due to multiple risk factors, in certain populations, such as that of LGTB people, which includes homosexual, bisexual, transsexual, intersexual and queer people, as well as any other gender identity, expression and sexual/romantic orientation (Biglia and Cagliero, 2019).

Focusing on sexual orientation, a historical-cultural overview of the perception (Foucault, 1980) and conception of homosexuality as a mental disorder (Ardila, 2022) shows that the attitudes toward this orientation have evolved. Their evolution seems to go in the same direction as that of racism and sexism, with the current differentiation between explicit and subtle attitudes (Rodríguez-Castro et al., 2013). Explicit homophobic attitudes include verbal, physical or psychological aggression toward homosexual people, whereas subtle homophobic attitudes include cultural or medical discourses about the pathological character of homosexuality and the incapacitation of homosexual couples to adopt children. For Kate Millett (Del-Olmo-Campillo, 2018), the rejection of homosexuality is a consequence of the oppression of a patriarchal state. Sexuality has a relevant role in social transformation. A revolution is necessary that makes us reconsider politically relationship between the sexes. A scenario in which sexuality occupies a free and priority role. Thus, social transformation can be achieved from a sexual revolution, one that eliminates strategies of domination and power present in sexual relations between men and women, and that achieves make all taboos and prohibitions relating to sexuality disappear, including homosexuality (Del-Olmo-Campillo, 2018).

In the European Parliament Resolution about homophobia in Europe, held in January 9th 2006 (Resolución del Parlamento Europeo sobre la homofobia en Europa, 2006), the concept of homophobia was defined as an irrational fear and aversion toward homosexuality and the LGTB community (lesbians, gays, bisexuals and transsexuals), based on prejudices and comparable to racism, xenophobia, antisemitism and sexism. In this resolution, it is considered that homophobia manifests in the public and private scopes in different forms, such as hate speech and encouragement of discrimination, ridicule, verbal, psychological and physical violence, prosecution and even murder. It has also been found that microaggressions can have an equally detrimental impact as explicit aggressions. Microaggressions are comments that are used in everyday life and may go unnoticed, but which equally denigrate, belittle and often insult, are so ephemeral and so normalized within language that they seem almost imperceptible. In the countries of the European Union, differences are observed in terms of tolerance towards the LGTB community. While in Spain, close to 70% of the Spanish population knows someone who is gay, lesbian or bisexual, and the Knowledge of transgender people has doubled in 7 years, now rising to 2 out of every 10 Spaniards; Italy and Poland, for example, are the European countries that they have less direct contact with LGTB people and less knowledge along with a greater denial (“non-existent” discrimination) about their situation of discrimination within the country, particularly in transgender and intersex people (Cantó and Arregui, 2022). In this sense, Poland, for example, has regulations such as the Equal Treatment Law. However, this is insufficient legislation since fight against discrimination reasons of sexual orientation only in the field of employment and vocational training. However, ethnic/racial and gender discrimination offers a broader scope (Bojarski, 2021).

Some studies (O’Hanlan et al., 1997) highlight that homophobia operates in two well-differentiated levels: internal and external. Internal homophobia represents the prejudices that all individuals internalise from their environment (family, school, religion, etc.). External homophobia is the open expression of such prejudices, which may range from social evasion or prohibition from the legal and/or religious perspective to all forms of violence.

Negative attitudes towards gays and lesbians have a great impact on these individuals, not only on a physical level, but also on a psychological level. For example, homosexual students have been reported to suffer from high levels of anxiety, somatic distress and even post-traumatic stress symptoms, situations that affect the quality of life of homosexual people (D’Augelli et al., 2002).

Despite the evidence about the negative repercussions of homophobic and lesbophobic attitudes on the quality of life of LGTB people, the WHO continues to report a lack of research and understanding regarding the health of this population, and it highlights the attitudes of civil workers (healthcare, education and social service professionals) toward LGTB people as significant barriers to solve the health disparities related to sexual minorities (Yingling et al., 2017). There is an increasing commitment in public healthcare to understanding and improving the health and welfare of this population, and it is especially important that professionals in the social, educational and healthcare sectors are adequately trained and informed to solve problems that persist in this group (White-Hughto et al., 2015; Thomas et al., 2017). In addition to solving problems, the professionals must provide support and, especially, empower individuals and communities.

Currently in Spain, progress has been made in equal treatment and opportunities for LGTB people and, especially, for trans people. This advance has been made possible thanks to the approval of Law 4/2023, for the real and effective equality of trans people and for the guarantee of the rights of LGTB people. This law represents a before and after in defining public policies aimed at preventing and acting against discrimination against LGTB people. However, there are many people and political parties with conservative ideology who are fighting to try to repeal it (Iglesias-Bárez, 2003).

Therefore, the present study was designed with the aim of identifying homophobic and lesbophobic attitudes in a Spanish young population, investigating the association of these attitudes with sociodemographic, cultural and political variables.

Regarding the hypotheses of this study:

Women are expected to have more positive attitudes than men.

Young people with higher levels of education are expected to have more positive attitudes.

Older people are expected to have more positive attitudes.

Heterosexual youth are expected to have more negative attitudes toward homosexual persons.

Young Catholics are expected to have more negative attitudes compared to those who consider themselves atheists or agnostics.

It is expected that the greater the inclination towards a more conservative political orientation, the more negative attitudes.

## 2 Materials and methods

### 2.1 Participants

A total of 325 young people participated in this descriptive, cross-sectional study, with an age range of 18–30 years (*xˉ* = 23.63; *σ* = 2.99) (65.8% women (*N* = 214) and 34.4% men (*N* = 111). With respect to sex and gender, participants self-identified as such. Table 1 shows the distribution of the sample with respect to the variables of education level, place of residence, political ideology, religion and sexual orientation.

**Table 1:** Distribution of the sample as a function of the independent variables.

Variables		*N (%)*
Education level	Compulsory secondary education	12 (3.7%)
	Higher secondary education	101 (31.1%)
	Higher vocational training	12 (3.7%)
	Medium vocational training	66 (20.3%)
	Higher education	134 (41.2%)
Place of residence*	City	193 (59.4%)
	Large town	81 (24.9%)
	Small town	51 (15.7%)
Political ideology	Far right	5 (1.5%)
	Right	32 (9.8%)
	Moderate	76 (23.4%)
	Left	195 (60.0%)
	Far left	17 (5.2%)
Religion	Catholic	165 (50.8%)
	Atheist	102 (31.4%)
	Agnostic	49 (15.1%)
	Other	9 (2.8%)
Sexual orientation	Heterosexual	240 (73.8%)
	Homosexual	47 (14.5%)
	Bisexual	38 (11.7%)

*Small town: 0–5,000 inhabitants; large town: 5000–10,000 inhabitants; city: over 10,000 inhabitants.

### 2.2 Procedure

The information was gathered using a questionnaire created with the Google Forms platform, which was administered online. The link to the questionnaire was disseminated in social networks and messaging apps, such as WhatsApp, Instagram and Facebook.

The participants were selected by convenience sampling, which consists in selecting the participants intentionally, based on their age and their willingness to participate in this study voluntarily, respecting their anonymity at all times. The snowball technique was also used, which is a non-probabilistic sampling method by which the selected participants recruit new participants from among the people they know. The dissemination of the questionnaire and the gathering of the data were carried out from August 15th 2020 to October 31st 2020.

The first page of the questionnaire contained written information about the in-formed consent, in which the participants agreed to participate in the study voluntarily. In addition to informing them about the anonymity of their answers, they were given contact details in case they needed to clarify any doubts. This study respects the ethical principles of the Declaration of Helsinki and was approved by the Human Research Bioethics Committee of the University of Almería (Spain) (registration code: 201699600000098).

### 2.3 Instruments

All participants completed a sociodemographic information gathering protocol that was designed *ad hoc* for this study, which collects information about the following variables: sex, education level, age, place of residence, political ideology, religion and sexual orientation.

Furthermore, the participants completed the Modern Homophobia Scale (Raja and Stokes, 1998). This instrument consists of two scales: one of them evaluates the attitudes toward gay people (22 items), and the other evaluates the attitudes toward lesbian people (24 items). Therefore, these scales measure homophobic and lesbophobic attitudes, respectively. In turn, each of these scales consists of three subscales: personal discomfort, deviation/changeability, and institutional homophobia. Personal discomfort measures direct attitudes toward homosexuality at the personal level. Deviation/changeability measures the conception of homosexuality from the psychological/biological perspective, considering it as a deviation from heterosexuality that can change. Institutional homophobia measures those attitudes that occur in the institutional scope. The scales have a Likert answer format from 1 (strongly disagree) to 5 (strongly agree). The lower the score, the more negative the attitude toward gays and lesbians. The reliability estimated through Cronbach’s alpha was 0.951 for the scale of homophobic attitudes, and 0.920 for the scale of lesbophobic attitudes (Raja and Stokes, 1998).

Example:

**Table:** 

Homophobic attitudes (MHS-G)					
Male homosexuality is a psychological disease.	1	2	3	4	5
Gay guys could be straight if they really wanted to.	1	2	3	4	5
I believe that marriage between two men should be legal.	1	2	3	4	5
Lesbophobic attitudes (MHS-L)					
Lesbians are incapable of being good mothers.	1	2	3	4	5
I do not mind seeing two girls holding hands.	1	2	3	4	5
I would not vote for a political candidate who declares herself to be a lesbian.	1	2	3	4	5

The scale that measures homophobic attitudes is structured as follows: items 1–9 measure personal discomfort; items 10–13 measure deviation/changeability; and items 14–22 measure institutional homophobia. This scale has been validated, presenting a Cronbach’s alpha of 0.927 for personal discomfort, 0.928 for deviation/changeability, and 0.847 for institutional homophobia. The scale of lesbophobic attitudes is structured as follows: items 1–11 measure institutional homophobia; items 12–21 measure personal discomfort; and items 22–24 measure deviation/changeability. The Cronbach’s alpha for this scale was 0.785 for institutional homophobia, 0.921 for personal discomfort, and 0.961 for deviation/changeability (Raja and Stokes, 1998).

Rodríguez-Castro et al. (2013) validated this instrument by subscales, obtaining in the subscale of attitudes toward gays a Cronbach’s alpha of 0.94, and 0.93 for the subscale of attitudes toward lesbian people.

León et al. (2017), in their adaptation of the Modern Homophobia Scale of Raja and Stokes, also validated this instrument, obtaining correlations between different items whose values ranged between 0.27 and 0.59 in the homophobia scale, and between 0.28 and 0.66 in the lesbophobia scale, presenting a high Cronbach’s alpha (0.89). In this study, the construct validity was also determined, showing a variance of 54.5% for the scale of homophobia toward homosexuals, and 54.3% for the scale of lesbophobia toward lesbians.

### 2.4 Data analysis

The data analysis was conducted using the SPSS v25 statistical software.

Firstly, a univariate descriptive analysis was performed, including the mean and standard deviation of the variable age, as well as percentages and frequencies of the variables sex, education level, political ideology, religion and sexual orientation.

Normality tests of the quantitative variables were used to determine whether we could apply parametric or nonparametric tests in subsequent analyses. In addition, since the number of data points used was greater than 50, the Kolmogorov–Smirnov statistic was selected for the normality tests. Since the normality tests for the quantitative variables showed a normal distribution, ANOVA and Student’s t were used for the tests of independence. The correlations between the different study variables were also analyzed using Pearson’s correlation tests.

The homophobic and lesbophobic attitudes were compared between men and women using a two-independent-means test, with Student’s t statistic. These attitudes were also compared through single-factor ANOVA as a function of education level, sexual orientation, religion and place of residence.

Lastly, to relate age and political ideology to the scores obtained in the scales and estimate correlation coefficents, Pearson’s correlation tests were carried out.

## 3 Results

### 3.1 Homophobic and lesbophobic attitudes as a function of sex

Table 2 shows the results obtained in the Modern Homophobia Scale for men and women. In this instrument, the lower the score, the greater the affectation of homophobic and lesbophobic attitudes presented by the person.

**Table 2:** Comparison of homophobic and lesbophobic attitudes between men and women.

Homophobic attitudes	Sex	*x̅ (S)*	*t*	*p*	*d*
Personal discomfort	Women	4.90 (0.37)	4.359	0.000	0.559
	Men	4.53 (0.86)			
Deviation/changeability	Women	4.90 (0.37)	2.998	0.000	0.381
	Men	4.63 (0.93)			
Institutional homophobia	Women	4.75 (0.37)	5.031	0.000	0.603
	Men	4.30 (0.90)			
Total scale	Women	4.84 (0.31)	4.592	0.000	0.603
	Men	4.45 (0.86)			

All the factors of the scale show more positive attitudes in women compared to men. Moreover, it was observed that the relationship of homophobic and lesbophobic attitudes with the variable sex was statistically significant (*t* = 4.592 and *p* < 0.001 in homophobic attitudes; *t* = 3.688 and *p* < 0.001 in lesbophobic attitudes) (Table 2).

### 3.2 Homophobic and lesbophobic attitudes as a function of education level

Table 3 presents the descriptive statistics (*xˉ* and *%*) and differences in the scales of homophobic and lesbophobic attitudes based on the education level of the participants. The single-factor ANOVA shows that there were significant differences in these attitudes as a function of the education level (*F* = 4.325 and *p* = 0.002 for homophobic attitudes; *F* = 3.946 and *p* = 0.004 for lesbophobic attitudes).

**Table 3:** Comparison of homophobic and lesbophobic attitudes as a function of education level.

	Education level	*x̅ (S)*	*F*	*p*	*η^2^*
Homophobic attitudes	Secondary education	4.13 (1.04)	4.325	0.002	0.051
	Higher secondary education	4.77 (0.46)			
	Medium VT	4.48 (0.41)			
	Higher VT	4.64 (0.74)			
	Higher education	4.76 (0.51)			
Lesbophobic attitudes	Secondary education	4.05 (0.82)	3.946	0.004	0.047
	Higher education	4.64 (0.46)			
	Medium VT	4.36 (0.37)			
	Higher VT	4.50 (0.71)			
	Higher education	4.61 (0.51)			

In the homophobic attitudes, the comparisons showed differences between the participants with compulsory secondary education and those with higher secondary education, higher vocational training and higher education. In the lesbophobic attitudes, differences were found between the participants with compulsory secondary education and those with higher secondary education and higher education. The participants with compulsory secondary education presented more negative attitudes, that is, the lower the education level, the greater the percentages of negative attitudes toward homosexual people (Table 3).

### 3.3 Homophobic and lesbophobic attitudes as a function of sexual orientation

Regarding the relationship of homophobic and lesbophobic attitudes with sexual orientation, significant differences were only identified in the lesbophobic attitudes (*F* = 4.571; *p* = 0.011) (Table 4).

**Table 4:** Comparison of the homophobic and lesbophobic attitudes as a function of sexual orientation.

	Sexual orientation	x̅ *(S)*	*F*	*p*	η^2^
Homophobic attitudes	Heterosexual	4.67 (0.66)	2.366	0.095	
	Homosexual	4.84 (0.19)			
	Bisexual	4.81 (0.36)			
Lesbophobic attitudes	Heterosexual	4.51 (0.62)	4.571	0.011	0.028
	Homosexual	4.73 (0.19)			
	Bisexual	4.71 (0.38)			

On their part, the comparisons showed differences between the participants with a heterosexual orientation and those with a homosexual orientation. The participants who identified themselves with a heterosexual orientation presented more negative attitudes (Table 4).

### 3.4 Homophobic and lesbophobic attitudes as a function of religion

Table 5 presents the descriptive statistics (*xˉ* and *%*) and the differences in the scales of homophobic and lesbophobic attitudes as a function of religion. There were significant differences in such attitudes based on religion (*F* = 6.142 and *p* = 0.002 for homophobic attitudes; *F* = 11.963 and *p* = 0.000 for lesbophobic attitudes).

**Table 5:** Comparison of the homophobic and lesbophobic attitudes as a function of religion.

	Religion	*x̅ (S)*	*F*	*p*	*η^2^*
Homophobic attitudes	Catholic	4.60 (0.73)	5.142	0.002	0.038
	Atheist	4.83 (0.33)			
	Agnostic	4.84 (0.36)			
Lesbophobic attitudes	Catholic	4.42 (0.68)	11.963	0.000	0.071
	Atheist	4.71 (0.34)			
	Agnostic	4.74 (0.35)			

In both attitudes, the comparisons showed differences between the Catholic participants and the Atheist and Agnostic participants. The Catholic participants presented more negative attitudes (Table 5).

### 3.5 Homophobic and lesbophobic attitudes as a function of the place of residence

Regarding the relationship between homophobic and lesbophobic attitudes as a function of the place of residence, no statistically significant differences were observed in any of the contemplated cases (*F* = 0.345 and *p* = 0.709 for homophobic attitudes; *F* = 0.893 and *p* = 0.410 for lesbophobic attitudes) (Table 6).

**Table 6:** Comparison of the homophobic and lesbophobic attitudes as a function of the place of residence.

	Place of residence	*x̅ (S)*	*F*	*p*	*η^2^*
Homophobic attitudes	Small town	4.65 (0.61)	0.345	0.709	
	Large town	4.71 (0.53)			
	City	4.72 (0.61)			
Lesbophobic attitudes	Small town	4.50 (0.60)	0.893	0.410	
	Large town	4.52 (0.52)			
	City	4.60 (0.56)			

### 3.6 Homophobic and lesbophobic attitudes as a function of age and political ideology

After investigating the relationship between homophobic and lesbophobic attitudes as a function of age, the Pearson’s correlation coefficients showed that age was negatively correlated with the scale of homophobic attitudes (*r* = −0.138; *p* < 0.05) and with the scale of lesbophobic attitudes (*r* = −0.131; *p* < 0.05). Therefore, the younger the participant, the more negative the attitudes presented by her/him.

It was also observed that political ideology was positively correlated with the scale of homophobic attitudes (*r* = 0.443; *p* < 0.01) and with the scale of lesbophobic attitudes (*r* = −0.131; *p* < 0.505). In participants with a left-wing ideology, more positive attitudes were observed toward gays and lesbians.

## 4 Discussion

The general aim of the present study was to identify homophobic and lesbophobic attitudes in a young Spanish population, analysing the relationship of these attitudes with sociodemographic, cultural, political, and personal variables. This study was conducted to respond to the discrimination of a minority group, i.e., people with a non-heterosexual orientation, whose quality of life may be negatively affected by certain explicit and implicit behaviours targeted to them (Yingling et al., 2017).

In this study, the male participants showed a more negative attitude toward homosexual people compared to the female participants. These results are in line with those of previous studies, which reported greater percentages of men than women with homophobic attitudes (Rodríguez-Castro et al., 2013; Borja-Gil and Núñez-Domínguez, 2014; Bosch, 2015; Penna-Tosso, 2015; Monzonís-Hinarejos, 2016). Moreover, this study detected that the men presented a more negative attitude toward lesbians compared to the women. This finding could be related to the behavioural roles and rules established by cultural norms. Men tend to internalise gender role norms more strongly than women. This would lead men to evaluate homosexual people more negatively, perceiving the latter as representatives of some sort of violation of the traditional norms of gender roles (Caycho-Rodríguez, 2010). In this sense, the consequences of a patriarchal system and toxic masculinity can be perceived.

Furthermore, Borja-Gil and Núñez-Domínguez (2014) detected that variables such as education level and age were also related to homophobic attitudes. Although there is a low correlation in this study, they found that younger and, therefore, less educated participants showed more intolerant attitudes toward homosexuality. It was also detected that, the greater the education level, the more positive the attitudes toward homosexual people. To validate these results, we could relate age to abstract thinking. Thus, the older the person, the greater their capacity for reciprocity in their relationships with other people, and the greater their capacity for critical thinking, reflection, and exploration (Melendres-Yallerco and Velarde-Torres, 2018).

Regarding the relationship of sexual orientation with homophobic/lesbophobic at-titudes, the present study demonstrated that the participants with a heterosexual orientation had more negative attitudes than those with a homosexual orientation. This result is in line with that found by other authors (España et al., 2001), who stated that the sociocultural influence could play an important role, since beliefs, values and traditional gender roles have an impact on the homophobia that heterosexual people develop. For instance, hypermasculinity is expressed and reaffirmed in men, and when a man moves away from the established parameters, the others may express and feel homophobia (Orcasita et al., 2020).

Moreover, some studies have shown that there is a greater percentage of Catholic people who present homophobic and lesbophobic attitudes compared to Atheists and Agnostics (Penna-Tosso, 2015; Monzonís-Hinarejos, 2016). The present study obtained results in the same line. Religion could contain a series of implicit requirements about the behaviours and roles expected of each gender, thus their transgression would be considered as a direct attack on these sacred beliefs (Barrientos-Delgado et al., 2014). However, Gastelo-Flores and Sahagún-Padilla (2020) observed in their study that discrimination against sexually diverse people was observed internationally; that is, in all cultures and religions. These findings suggest a socialization of heterosexuality. In other words, a subtle way of imposing a specific sexual orientation, which could give rise to homophobic situations in part of the population internationally (Gastelo-Flores and Sahagún-Padilla, 2020).

Concerning the political ideology variable, this study detected that, the greater the tendency toward right-wing ideologies, the greater the percentage of negative attitudes toward LGTB people. This result is consistent with those found by Rottenbacher (2012), who identified a positive correlation between conservatism and homophobic behaviours, as well as with the conclusion of the meta-analysis of Penna-Tosso (2015). Most people with right-wing and extreme right-wing ideologies present a high degree of adherence to the rules that support specific ideas about what they consider to be “normal,” i.e., to conservative (hetero) normative dictates. Right-wing ideologies, and especially extreme right-wing ideologies, do not usually adhere to rules based on acceptance or civil human rights. In addition, a strong relationship has been found between value conservatism and identifying oneself with traditionalist ideas and support for right-wing parties (Barrientos and Cárdenas, 2013). In this sense, the progress of all political parties towards recognition of gender and sexuality diversity is of vital importance. This situation would facilitate an increase in tolerance towards the LGTB community in many people of different ideologies. In addition, more public educational policies could be carried out to facilitate the visibility of education for equal opportunities in educational centers. This situation, in turn, could deconstruct all types of prejudices, discrimination and violence, especially regarding issues of gender and sexuality (Souza and Fialho, 2020).

Lastly, with respect to the place of residence and its relationship with negative attitudes toward homosexual people, this study did not detect differences in such attitudes in the participants as a function of their place of residence. This result is in line with that reported by Tate (1991). However, other studies confirm that living in a large city could be a protective factor, as it provides people with better social support networks, which allow them to better avoid the negative effects of homophobia (Institute of Medicine, 2011; Barrientos-Delgado et al., 2014). This social support networks are one of the most important protective factors for the LGTB community.

### 4.1 Limitations

This study presents important strengths. First, the veracity of the hypotheses put forward has been demonstrated. Moreover, very useful and updated information was generated on a set of indicators of homophobia and lesbophobia in a young population in Spain, thereby contributing to future research lines in the approach of this problem from current data. This fact shows that even though Spain is one of the European countries which is making progress in the recognition of the human rights of the LGTB community, and that it is the fourth European country in terms of LGTB rights, there are still homophobic and lesbophobic attitudes that may pose a problem for the well-being of homosexual people. Based on our current results, it would be interesting to draw up a programme to raise awareness in society about the consequences that certain negative attitudes may have on the LGTB community, and not only on homosexual men and women. Furthermore, a possible roadmap is set for future, larger investigations in this line. However, this study also presents some limitations that must be pointed out.

One of the weaknesses of this research is that other negative attitudes towards other LGTB populations, such as bisexual, transgender, or intersex people were not studied. It would be interesting to carry out further research in order to detect certain biphobic or transphobic attitudes since this part of the community, i.e., gender non-conforming, transgender, or intersex people are still invisible in this type of research. Additionally, future research could also incorporate qualitative methods to gain deeper insights into personal experiences and social influences on homophobic and lesbophobic attitudes.

Due to the complications generated from the circumstances that took place during the field work, such as the SARS-CoV-2 pandemic, the questionnaire was administered online, which limited the number of people and specific population groups that could be reached, as some people still lack access to social networks. It is also important to remark that the questionnaire also reached out to more conservative people who refused to participate in the research, thus presenting a negative attitude towards issues related to homosexuality. In addition, it is also acknowledged that some groups were small as well as some effect sizes, which can be related to the type of sampling used. This limited the representativeness of the sample and therefore the chances of obtaining more representative results. It would be recommended that future research on this topic be carried out with random sampling techniques and with larger samples in different countries. In this way, the potential to extrapolate results would increase.

## 5 Conclusion

This study shows the relationship of certain sociodemographic, political, personal and cultural variables with homophobic and lesbophobic attitudes. It is shown that some of the categories of these variables may negatively influence gay men and lesbian women.

This study also reports different scores in the results obtained according to sex, which corroborates the importance of considering the gender perspective in future studies. Here we can consider the effects of toxic masculinity, which has to be taken into account as a factor for future research, since it is not only the fact of being a woman or a man that affects homo/lesbophobia, but also the ideals of masculinity to which men adhere.

On the other hand, in today’s society homogeneous heterocentrism is still preserved, where the leader of the nation is analogous to the father in the traditional patriarchal family. Therefore, it is necessary to carry out research and interventions on gender and cultural construction of human sexuality in secondary education (Stanley, 2018).

In short, the present work highlights the need to use these results from a psychosocial and educational approach to modify vulnerable targets through the design and implementation of training and preventive programs. This will exponentially improve the quality of life of homosexual persons.

## Data availability statement

The raw data supporting the conclusions of this article will be made available by the authors, without undue reservation.

## Ethics statement

The studies involving humans were approved by Human Research Bioethics Committee of the University of Almería (Spain) (registration code: 201699600000098). The studies were conducted in accordance with the local legislation and institutional requirements. The participants provided their written informed consent to participate in this study.

## Author contributions

J-RG-S: Writing – review & editing, Writing – original draft. F-JG-V: Writing – review & editing, Writing – original draft. M-JL-L: Writing – review & editing, Writing – original draft. AS-C: Writing – review & editing, Writing – original draft. L-CS-d-l-T: Writing – review & editing, Supervision, Formal analysis, Funding acquisition.EM-S: Writing – review & editing, Writing – original draft.
